# The miR-166a-3p in exosome-like nanoparticles from *Stellera chamaejasme* induce insecticidal effects in fall armyworm by disrupting the gut barrier and microbiota

**DOI:** 10.3389/fpls.2026.1902418

**Published:** 2026-09-30

**Authors:** Liwei Zhang, Teng Zhao, Lidan Lu, Ruyi Li, Wentao Gu, Liang Zou, Lianxin Peng, Kayvan Etebari, Yuanhang Ren

**Affiliations:** 1 Key Laboratory of Coarse Cereal Processing, Ministry of Agriculture and Rural Affairs, Sichuan Engineering and Technology Research Center of Coarse Cereal Industrialization, College of Food and Biological Engineering, Chengdu University, Chengdu, China; 2 School of Agriculture and Food Sustainability, The University of Queensland, Gatton, QLD, Australia

**Keywords:** *Stellera chamaejasme*, exosome-like nanoparticles, microRNA, fall armyworm, midgut, gut microbiota

## Abstract

Plant-derived exosome-like nanoparticles (ELNs) are a class of extracellular vesicles that have been shown to exert cross-species regulatory effects. This provides a new strategy for discovering novel insecticidal components in plants. In our study, the *Stellera chamaejasme*-derived exosome-like nanoparticles (Sc-ELNs) were first isolated and characterized. The insecticidal activity assays showed that the Sc-ELNs and miRNAs in it both exhibit inhibitory activity against fall armyworm (FAW) *in vivo*. It is worth noting that miR-166a-3p agomir can affect the food digestive absorption function of FAW by inducing apoptosis of intestinal epithelial cells and reducing the abundance of gut bacteria associated with polysaccharide utilization. Concurrently, multiple tricarboxylic acid (TCA) cycle-related pathways in bacteria were inhibited by miR-166a-3p agomir. Overall, this study demonstrates that Sc-ELNs and miR-166a-3p in it possess potential insecticidal effect, which provided a new pesticidal perspective for *S. chamaejasme*.

## 1 Introduction

*Stellera chamaejasme* L. (Thymelaeaceae, Malvales) is a perennial weed with a wide geographical distribution in China, Mongolia and the siberian region of Russia (Li et al., 2018). It is described in the Shennong’s Classic of Materia Medcia, an early Chinese herbal medicine text, as a traditional herbal remedy with anti-inflammatory properties. Recent studies have also shown that *S. chamaejasme* exhibits pronounced insecticidal, acaricidal and nematicidal activities (Jin et al., 2018). Research on bioactive constituents of *S. chamaejasme* indicates that its insecticidal activity is primarily associated with biflavonoids, including neochamaejasmin A (NCA), neochamaejasmin B (NCB), and isochamaejasmine (ICM) (Ren et al., 2019, Ren et al., 2021a, Ren et al., 2021b; Gu et al., 2022). Fall armyworm (FAW; *Spodoptera frugiperda*) is one of the most serious agricultural pests worldwide. In our previous study, ICM at 2 mg/mL caused >90% mortality in FAW within 96 hours. Moreover, ICM and NCA can induce mitochondrial dysfunction and trigger programmed cell death in FAW ovarian cells (He et al., 2025). These findings highlight *S. chamaejasme*, as a non-FAW host plant, is a promising source of plant-derived insecticidal agent for FAW management. However, despite the identification of several active ingredients, it remains unclear whether *S. chamaejasme* also contains additional classes of insecticidal substances, particularly compounds with modes of action distinct from those of well- characterized phytochemicals.

Exosomes are a class of extracellular vesicle (EV) released when multivesicular bodies (MVBs) fuse with the plasma membrane. They were first observed in cultured sheep reticulocytes and were subsequently termed “exosomes” in 1987 (Edgar, 2016; Johnstone et al., 1987). Exosomes are typically 30–150 nm in diameter and are enriched in bioactive molecules, including proteins, lipids, and nucleic acids such as mRNA, miRNA, and DNA (Kalluri and LeBleu, 2020). By delivering biomolecular cargo, exosomes mediate long-distance intercellular communication. Among their cargo, miRNAs have attracted particular attention due to their key roles in post-transcriptional gene regulation (Joshi et al., 2020; Nail et al., 2023). miRNAs are 18–24 nt noncoding small RNAs that serve as key post-transcriptional regulators in both plants and animals (Li et al., 2021). By base-pairing with complementary sites on target mRNAs in the host organism, they repress translation or promote mRNA degradation and thereby influencing cell differentiation, proliferation, development, immune responses, and apoptosis (Li et al., 2021).

Recent studies indicate that exosomes not only mediate miRNA transfer and regulation between cells within a host, but can also transmit miRNAs across species and modulate gene regulation in recipient organism (Li et al., 2022). For example, the ginger-derived ELN markedly suppress NLRP3 inflammasome activation in mouse bone marrow-derived macrophages (BMDMs), and its cargo, including osa-miR164d, can alleviate colitis-related symptoms in mice (Yan et al., 2025). Similarly, garlic chive-derived ELNs likewise inhibit NLRP3 activation in BMDMs and attenuate inflammation in chemically induced acute liver injury (Liu et al., 2021). In addition, the grapefruit-derived nano vectors (GNVs) loaded with miR-17 could suppress brain tumor growth in mice (Zhuang et al., 2016). Overall, these studies suggest that ELNs and their miRNAs cargo can contribute to cross-species regulation. This phenomenon provides a potential avenue for discovering new categories plant-derived insecticidal agents. Accordingly, we hypothesize that *S. chamaejasme* produces ELNs carrying miRNAs that could modulate physiological processes in FAW, thereby impacting its survival and growth.

In this study, we first isolated and identified Sc-ELNs from *S. chamaejasme*, and then analyzed the miRNA cargo in Sc ELNs, further predicting the potential binding genes of these miRNAs in insects. Then, we systematically evaluated the *in vitro* and *in vivo* insecticidal activity of Sc ELNs and miRNA agomirs against FAW. And the effects of miR-166a-3p agomir on intestinal integrity and gut microbiota in FAW were studied. The results showed that Sc-ELNs caused mortality in FAW and inhibited SF9 cell proliferation. Additionally, the miR-166a-3p agomir reduced larval weight and length, effects that were associated with midgut barrier damage and disruption of gut microbiota homeostasis. Overall, these findings reveal the presence of a new potential insecticidal active substance in *S. chamaejasme* which provided a new pesticidal perspective for it.

## 2 Materials and methods

### 2.1 Plant materials and reagents

The *S. chamaejasme* was collected from Ruoergai County, Sichuan Province, China, and was washed and air-dried at room temperature prior to use. The fourth-instar fall armyworm (FAW) larvae were purchased from Jiyuan Baiyun Industrial Co., Ltd. (Henan, China) and reared at 25 ± 1 °C with 75 ± 1% relative humidity under a 16:8 h (light: dark) photoperiod, and fed fresh maize kernels. The main reagents used in this study included polyethylene glycol 6000 (PEG-6000), phosphate-buffered saline (PBS), and RNase-free water, all purchased from Solarbio (Beijing, China). All other reagents were of analytical grade (AR) and obtained from Servicebio (Wuhan, China).

### 2.2 Extraction of Sc-ELNs via ultracentrifugation

The extraction procedure was optimized based on previously reported methods (Mendonca et al., 2025). The operation steps are shown in **Figure 1A**. The 10 g dry root tissue of *S. chamaejasme* was cut into small pieces, surface sterilized by 75**%** (v/v) ethanol and sectioned with a sterile scalpel, thoroughly rinsed with distilled water, and blotted dry with filter paper. The tissue was enzymatically digested at 50 °C for 6 hours in a 50 mL solution containing 4**%** (w/v) cellulase (S10041, Shanghai Yuanye Co., Ltd), 2**%** (w/v) pectinase (S10007, Shanghai Yuanye Co., Ltd), and 0.6 mol/L mannitol (A600335, Shanghai Shenggong Co., Ltd) with a pH of 5.8 (adjust with NaOH/HCl).

**Figure 1:**
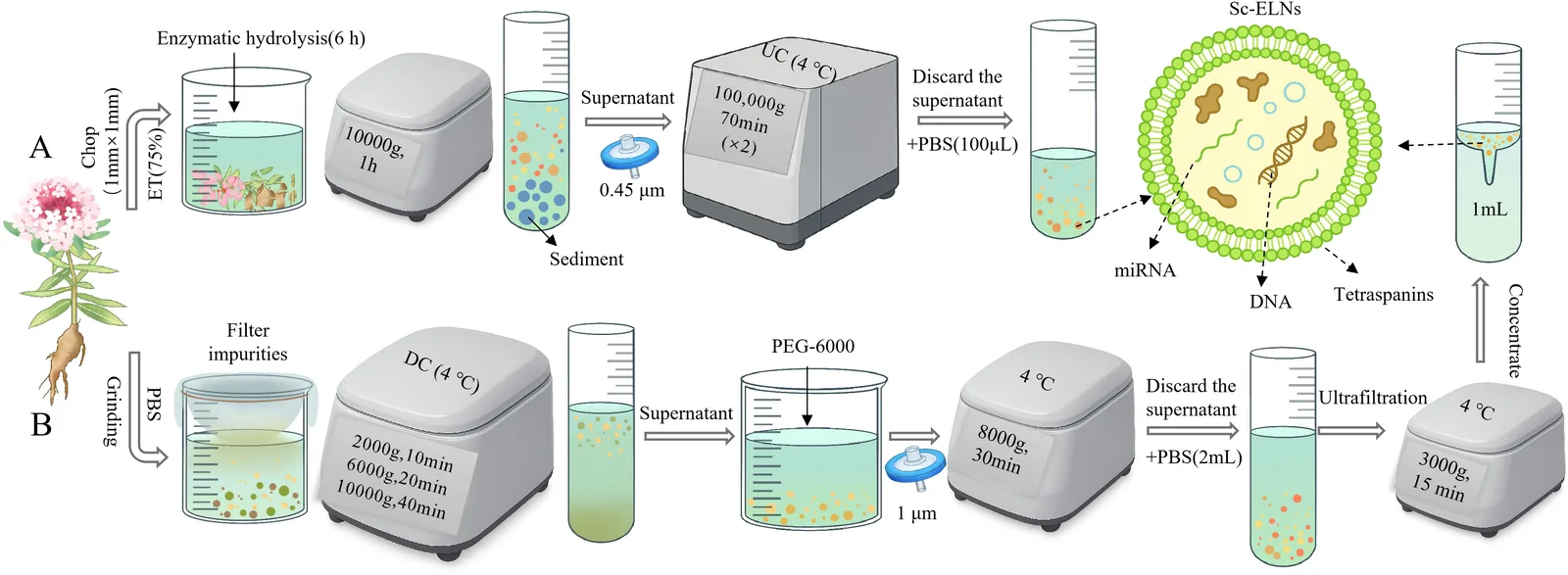
Different extraction methods for Sc-ELNs. Ultracentrifugation **(A)** and PEG precipitation **(B)**. UC, ultracentrifugation; DC, differential centrifugation.

After digestion, the suspension was centrifuged at 10,000 × g for 1 h to remove tissue debris, and then passed through a 0.45 μm membrane filter to remove larger particles. The filtrate was transferred into ultracentrifuge tubes and centrifuged at 100,000 × g for 70 min at 4 °C. The pellet was resuspended in 10 mL of pre-chilled PBS and ultracentrifuged again under the same conditions. After discarding the supernatant, the final pellet (Sc-ELNs) was resuspended in 100 µL of cold PBS.

### 2.3 Extraction of Sc-ELNs via PEG-based precipitation

The extraction procedure was optimized based on previously reported methods (Ban et al., 2015). The workflow is summarized in **Figure 1B**. Briefly, the 10 g dry root tissue of *S. chamaejasme* was cut into small pieces, mixed with 50 mL pre-chilled PBS. Then, the mixture was poured into a homogenizer and homogenized for 1 min. This step was repeated five times to obtain a homogenate. Crude debris in homogenate was removed by filtration through medical gauze. The filtrate was kept at 4 °C and subjected to differential centrifugation at 2,000 × g for 10 min, 6,000 × g for 20 min, and 10,000 × g for 30 min. The resulting supernatant was added to 15% (w/v) PEG-6000 and incubated overnight at 4 °C. Then the samples were passed through 1 µm membrane filter and centrifuged at 8000 × g for 30 min at 4 °C. The pellet (Sc-ELNs) was resuspended in 2 mL PBS, and further concentrated using an ultrafiltration device by centrifugation at 3,000 × g for 15 min at 4 °C.

### 2.4 Characterization of Sc-ELNs

#### 2.4.1 Transmission electron microscope analysis

TEM analysis was performed according to previously published methods (Quan et al., 2020). Briefly, 2 µL of sample was diluted with PBS to 10 µL, applied it to a copper grid, and allowed to adsorb for 1 min. Excess liquid was removed with filterpaper, the grid was negatively stained with 10 μL of 2% uranyl acetate for 1 min. The stain was blotted off and the grid was air-dried at room temperature for several minutes before imaging. Samples were imaged using a Hitachi HT7800 transmission electron microscopy (Hitachi, Japan).

#### 2.4.2 Nanoparticle tracking analysis

Sc-ELNs were diluted in sterile buffer prior to analysis (10 μL brought to a final volume of 30 μL) (Sha et al., 2024a). Instrument performance was verified using calibration standards before sample measurement. Particle size distribution and particle concentration were measured using an NTA instrument (NanoSight N30E, Malvern Panalytical, United Kingdom) and analyzed with the manufacturer’s software (van der Pol et al., 2014).

### 2.5 miRNA sequencing analysis

Total RNA was isolated by the kit (DP419, Beijing Tiangen Co., Ltd) and used for small RNA library preparation following standard protocols (Liu et al., 2022). After 16 cycles of PCR enrichment, DNA fragments corresponding to 140–160 bp were size selected by gel electrophoresis, followed by library quality assessment and resuspension in ultrapure water. Sequencing was performed on an Illumina HiSeq 2500 system. Raw image data were converted to sequence reads by base calling. Adapter and primer sequences were trimmed from the FASTQ files, and reads were then quality filtered (obtaining clean reads) and size selected (21–22 nt). The miRNA abundance was estimated as transcripts per million miRNA reads (TPM).

### 2.6 miRNA target gene prediction

The *Drosophila melanogaster* genomes (GCF_000001215.4) were downloaded along with the sequence information of miRNAs identified from Sc-ELNs sequencing (this study) from NCBI. Putative miRNA target genes were predicted using Miranda (parameter settings: -sc 140 -en -10- scale 4 -step) and RNAhybrid (parameter settings: [-e energy_cutoff] -10 [-p p-value_cutoff] 0.05 [-m max_target_length] 50000) which evaluate sequence complementarity and binding free energy. Using these two softwares to independently predict and select the consistent results as the targeted mRNA of the host by miRNA, and to further determine the targeted host DNA through mRNA analysis (Etebari and Asgari, 2016, Etebari and Asgari, 2014). Predicted targets were then subjected to Gene Ontology (GO) and Kyoto Encyclopedia of Genes and Genomes (KEGG) enrichment analyses by diamond v0.8.22 and KEGG Automatic Annotation Server (Ashburner et al., 2000; Kanehisa et al., 2021).

### 2.7 Insecticidal activity of Sc-ELNs and miRNAs

#### 2.7.1 FAW

Sc-ELNs were prepared as described in section 2.3. The miRNA agomirs was synthesized by Qingke Biotechnology (Beijing, China). The agomirs used in this study is a class of double-stranded miRNA analogs modified with full-length 2’-O-methoxy, terminal phosphorothioate and cholesterol groups. Compared with conventional miRNA mimics, it has significantly improved nuclease degradation resistance and tissue penetration, and can stably mediate the target gene silencing effect of miRNA in complex biological systems. The impact of Sc-ELNs and miRNA agomirs on FAW larvae was assessed by intrahemocoelic injection (Shen et al., 2020). Briefly, 1 μL Sc-ELNs (9.0 × 10¹¹ particles/mL) or miRNA mimic (100 μM) was injected into the hemocoel of 4^th^ larvae using a microsyringe via the abdominal proleg. Control received 1 μL of PBS (for Sc-ELN treatments) or nuclease-free water (for miRNA treatments). Each treatment included four independent replicates, with 10 larvae per replicate. Mortality was recorded at indicated times and corrected using Abbott’s formula: Corrected Mortality (%)=(Mt - Mc)/(1 - Mc) × 100, where Mt and Mc are the observed mortality proportions in the treatment and control groups, respectively (Abbott, 1925). Larval weight, length, and food intake were recorded daily at same timepoint. The dead larvae on that day need to be included in the daily statistics and discarded afterwards. Weight: Larvae in each treatment were weighed as a whole using an electronic balance (FA1204C, Shanghai Yueping Instrument Co., Ltd., China), and the average individual weight was calculated by dividing the total weight by the number of larvae. Length: Larvae were photographed alongside a ruler under a stereomicroscope (SZX7, Olympus, Japan), and body length was subsequently measured from the digital images. The daily food intake per insect was calculated by weighing the total fresh corn kernels provided to each group prior to feeding, evenly distributing them among the tested insects, and subsequently weighing the remaining kernels collected the following day. The difference between the initial and remaining weights was divided by the number of insects in each group to obtain the average individual food consumption.

#### 2.7.2 Sf9 cells

The MTT assay was used to evaluate the antiproliferative effect of Sc-ELNs on Sf9 cells (ovarian cells of FAW) (He et al., 2020). Sf9 cells in logarithmic growth phase were seeded in 96-well plates at 1.0 × 10^5^ cells per well in 100 μL of culture medium (Procell, Wuhan, China) and incubated overnight. The medium in the treatment wells was then replaced with Sc-ELNs (9.0 × 10¹¹ particles/mL) diluted 1:1 (v/v) in fresh medium. Control wells received PBS diluted 1:1 (v/v) in fresh medium. Blank wells filled with medium without cells. Each experimental group is set up with 5 parallel wells. Cell proliferation inhibition was calculated as (OD _control_-OD _treatment_)/OD _control_ ×100% after three biological replicates.

### 2.8 Histopathological analysis of the midgut via hematoxylin-eosin staining

The miRNA treatment concentration and exposure time was followed to the procedure described in Section 2.7.1. Midgut tissues were dissected from FAW larvae in the miR-166a-3p agomir-treated and controls group after 96 hours of rearing. Tissue was rinsed in pre-chilled PBS and fixed in 4% paraformaldehyde (Servicebio, Wuhan, China) at 4 °C for 24 h. The fixed samples were then paraffin embedded and sectioned at 4 μm. Sections were stained with hematoxylin (Servicebio, Wuhan, China) for 5 min, dehydrated with 95% ethanol for 1 min, and counterstained with eosin for 15 s, according to the manufacturer’s instruction. Afterwards, the slices were sequentially dehydrated in a gradient of anhydrous ethanol and n-butanol provided by the manufacturer (2 minutes each time, a total of 5 times). Transparent slices were made using xylene and sealed with neutral gum. The histopathological changes in the midgut were examined using a light microscope (Nikon, Japan).

### 2.9 Ultrastructural analysis of the midgut by TEM

Midgut treatment and dissection were performed as described in Section 2.8. Dissected FAW midgut tissue was fixed in 2.5% glutaraldehyde at 4 °C for 24 h. The samples were washed three times with PBS. Subsequently, they were dehydrated in a graded ethanol series (30–100%), and then in 100% acetone as a transitional solvent. Tissues were embedded in Epon 812 and sectioned into ultrathin slices (60–80 nm) by ultramicrotome. Sections were stained with 2% uranyl acetate for 8 min and 2.6% lead citrate for 8 min, rinsed with ultrapure water, air-dried overnight at room temperature, and placed on a copper grid. Ultrastructural features of the midgut were examined using a transmission electron microscope (HT7800, Hitachi, Japan).

### 2.10 Apoptosis analysis

#### 2.10.1 TUNEL assay

Midgut treatment, dissection, fixation, paraffin embedding, and sectioning were performed as described in Section 2.8. Paraffin-embedded sections were deparaffinized and rehydrated, then processed for protease digestion using 20 μg/mL protease K according to the TUNEL kit instructions (Servicebio, Wuhan, China). Then the sections incubated at 37 °C for 20 minutes, then wash 3 times with PBS to completely remove proteinase K. The 50 μL equilibration buffer (Servicebio, Wuhan, China) was added on sections and incubated at room temperature for 20 minutes. Sections were incubated with the TdT reaction mixture (1 μL TdT, 5 μL FITC-dUTP, 50 μL equilibration buffer) at 37 °C for 1 h in the dark, washed with PBS, and counterstained with DAPI (Servicebio, Wuhan, China). Sections were washed with PBS and mounted with an anti-fade reagent. The FITC (excited at 488 nm and detected at 500–550 nm) and DAPI (excited at 405 nm and detected at 425–475 nm) signals were imaged using fluorescence microscope (E200, Nikon, Japan).

#### 2.10.2 Immunohistochemistry

Midgut tissue preparation (treatment, dissection, fixation, paraffin embedding, and sectioning) followed Section 2.8. Midgut sections were baked at 60 °C for 1 h, deparaffinized in xylene, and rehydrated through graded ethanol. Antigen retrieval was performed with EDTA buffer (pH 9.0), and endogenous peroxidase activity was blocked with 3% H_2_O_2_. Sections were blocked with goat serum and incubated with anti-Bax primary antibody (dilution ratio is 1:50) (SC-7480, Santa Cruz, USA) at 37 °C for 1 h, washed with PBS and followed incubation with HRP-labeled secondary antibody (dilution ratio is 1:500) (Kit-5230, Maixin, China) at room temperature for 20 minutes. After washed with PBS, the signal was developed using 3,3′-diaminobenzidine (DAB), and section were counterstained with hematoxylin, dehydrated, and mounted. Bax staining was examined using a light microscope (E200, Nikon, Japan).

### 2.11 Metagenomic analysis of gut bacteria in FAW

miRNA treatment and gut dissection were performed as described in Section 2.8, with all dissection conducted under sterile conditions. Each treatment included three independent replicates, and with 20 larvae per replicate. The dead larvae were collected and frozen in a timely fashion. After the 96 hours, the all guts of each group were pooled, and total DNA was extracted and quantified. DNA was sheared to an average insert size of ~350 bp and used for libraries construction, including end-repair, A-tailing, adapter ligation, and PCR amplification. Library fragment size was verified and libraries were quantified by qPCR (>3 nM). Equimolar libraries were pooled and sequenced on an Illumina NovaSeq 6000 platform. Raw reads were processed with fastp (v0.23) to remove adapters, low-quality bases (Q ≤ 5, >50%), and reads containing >10% Ns. Host-derived reads were removed by alignment to *S. frugiperda* genome (GCF_023101765.2) with Bowtie2. Clean reads were co-assembled with megahit v1.2.9 with default parameters. Scaffolds were split at N positions to generate scaftigs. The ORFs were predicted from scaftigs ≥500 bp for each sample using MetaGeneMark. Clean reads were mapped back to the predicted genes using Bowtie2, and gene abundance was estimated as relative abundance, which calculated from the number of mapped reads and the corresponding gene length (Cotillard et al., 2013). Representative unigenes were assigned taxonomic annotation using Micro_NR database. Functional annotation was performed by aligning unigenes against the Micro_NR, CAZy and KEGG database using diamond v0.8.22 (Buchfink et al., 2014; Karlsson et al., 2013). The statistical analysis of species and functional differences between groups was conducted using MetaGenomeSeq analysis, the * represents P adj < 0.05.

### 2.12 Data analysis

All experiments were performed with at least three independent biological replicates, and data are presented as the mean ± standard error. Statistical analyses were performed using IBM SPSS 19.0 (IBM, USA). Comparisons among multiple groups were analyzed using one-way analysis of variance (ANOVA) followed by Tukey’s multiple-comparison test. Pairwise comparisons were analyzed using Student’s t- tests. Graphs were generated using GraphPad Prism 10.4.0 (GraphPad Software, San Diego, CA, USA). Statistical significance was defined as p < 0.05 (*) and p < 0.01 (**).

## 3 Results

### 3.1 Characterization of Sc-ELNs

Sc-ELNs were isolated using both ultracentrifugation and PEG-based precipitation. TEM images showed vesicle-like particles with a cup-shaped morphology (**Figures 2A, C**). NTA indicated that ultracentrifugation-derived Sc-ELNs had a relatively uniform narrow size distribution, with a mean diameter of 77 nm and a concentration of 4.9 × 10^9^ particles/mL (**Figure 2B**). In contrast, PEG-precipitated Sc-ELNs displayed a larger mean diameter (238 nm) and a substantially higher particle concentration of 9.0 × 10¹¹ particles/mL (**Figure 2D**). The nanoparticles obtained from *S. chamaejasme* using two different extraction methods both exhibited typical morphological characteristics and particle size of exosomes, indicating the presence of exosomes in the *S. chamaejasme.*

**Figure 2:**
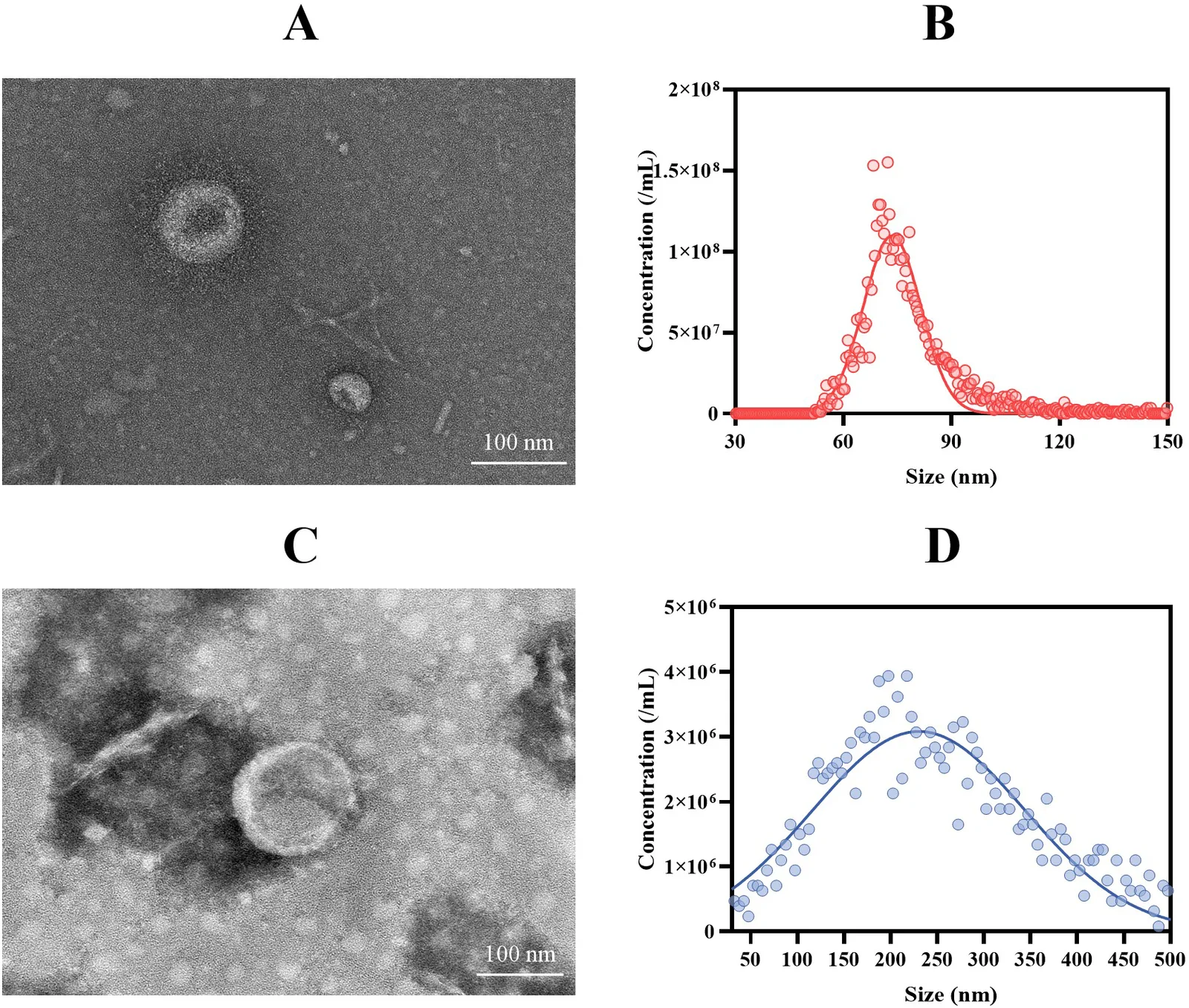
Morphological characterization of Sc-ELNs obtained by different extraction methods. TEM image of Sc-ELNs extracted by ultracentrifugation **(A)** and PEG precipitation **(C)**. Particle size of Sc-ELNs extracted by ultracentrifugation **(B)** and PEG precipitation **(D)**.

### 3.2 Target prediction of miRNAs

Due to the main role of miRNAs in the cross-species regulation of ELNs, the sRNA component of Sc-ELNs were further analyzed (**Supplementary Table S1**). After comparison with the mature miRNA sequences in miRBase database, five mature miRNAs were identified from Sc-ELNs (**Table 1**). Each miRNA was 21 nucleotides long, with GC contents ranged from 38.09 to 61.90%. The expression levels ranged from 83,333 to 250,000 TPM. Using miranda and RNAhybrid, these miRNAs were predicted to collectively target 23 genes in *D. melanogaster*.

**Table 1:** Prediction of target genes of the miRNAs in *Drosophila*.

miRNA ID	Expression level (TPM)	Sequence	Length	GC content (%)	Target gene
miR159a	250000	UUUGGAUUGAAGGGAGCUCUA	21	42.85	*Mtch, Cyp4s3, dpr19, CG15096, IntS8*, *Dh44-R1, CG43116, Rab8*
miR159b-3p	250000	UUUGGAUUGAAGGAGCUCUU	21	47.61	*Mtch, Cyp4s3, robl37BC, CG15096, IntS8*, *Dh44-R1, CG43116, Rab8, dpr19*
miR159c	166666	UUUGGAUUGAAGGGAGCUCCU	21	47.61	*CG18508, Mtch, COX7B, CG15096*, *CG43116, Rab8, lov*
miR166a-3p	250000	UCGGACCAGGCUUCAUUCCCC	21	61.90	*AnxB10, Snx27, MFS1, Mur2B*, *mrva, Mal-B2*
miR396b-5p	83333	UUCCACAGCUUUCUUGAACUU	21	38.09	*Osi, Lkb1, CG14257, Lbl, Lmpt*

Several predicted targets are linked to mitochondrial function and cellular metabolism. For example, *Mtch* encodes a mitochondrial carrier protein associated with mitochondrial function, apoptosis, and energy metabolism. *MFS1* is predicted to encode a transporter involved in movement of sugars, organic acids, and small metabolites across cellular membranes. *Mal-B2* encodes a maltase involved in maltose hydrolysis, contributing to carbohydrate digestion and energy metabolism. Another predicted target gene is *Lkb1*, which encodes a serine threonine protein kinase that acts as a central regulator of cellular energy metabolism, *Lbl* encodes a lipid-binding protein involved in lipid transport and storage. Overall, the predicted miRNA target genes are enriched for genes involved in mitochondrial function and substrate transport, suggesting that Sc-ELNs may interfere with insect physiology by disrupting energy metabolism.

GO term enrichment analysis of the predicted target genes highlighted terms including cell cortex part, cell death, apoptosis, programmed cell death, cellular carbohydrate metabolism, response to drug, and sucrose metabolic process (**Figure 3A**). KEGG pathway analysis similarly highlighted pathways associated with programmed cell death and energy metabolism, including autophagy, apoptosis, and starch and sucrose metabolism. Together, these enrichment results suggest that Sc-ELN miRNAs may influence pathways related to programmed cell death and energy metabolism, although these predicted interactions require experimental validation in FAW (**Figure 3B**).

**Figure 3:**
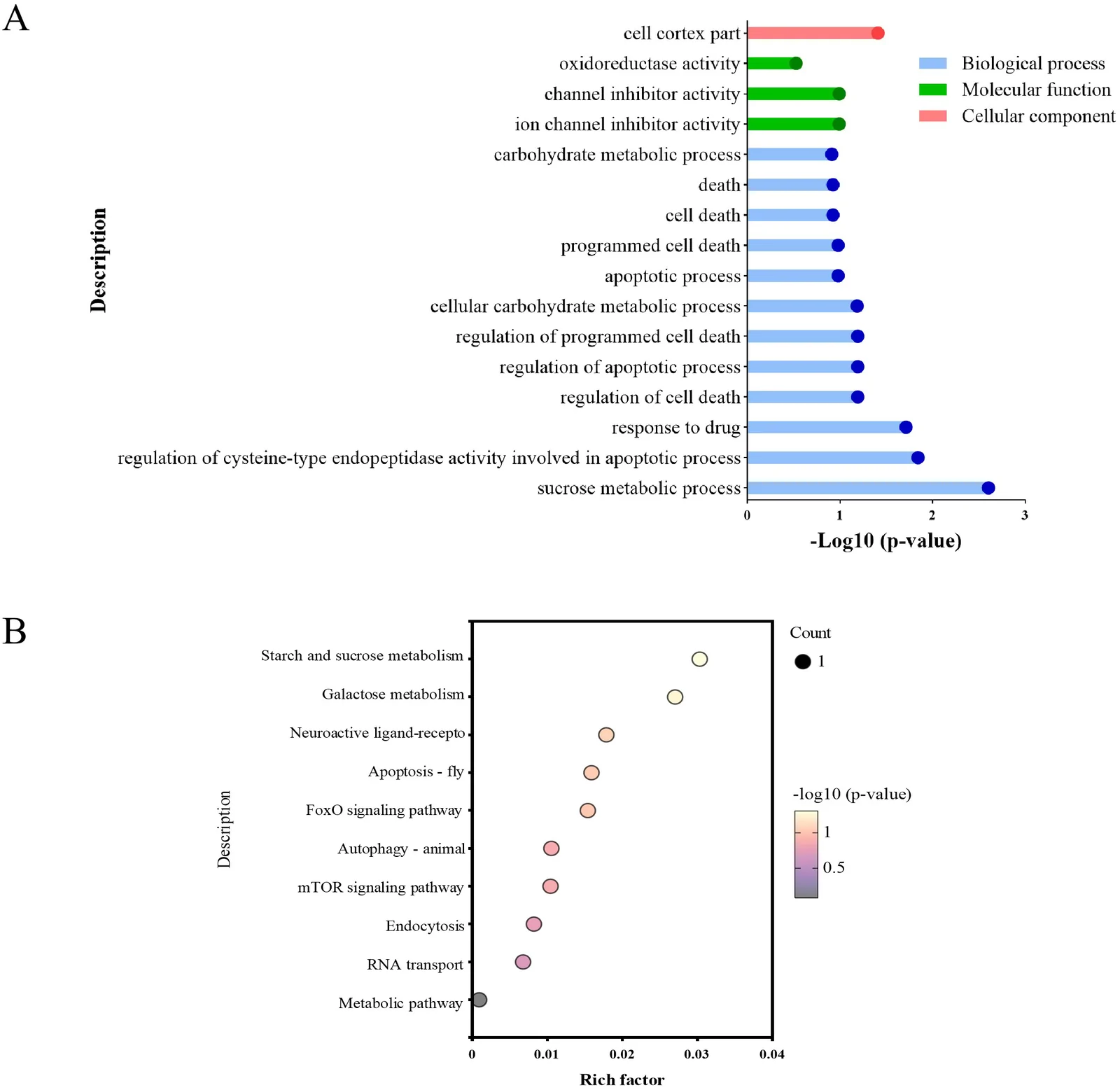
The function analysis of target gene for miRNA in Sc-ELNs. GO enrichment **(A)** and KEGG enrichment **(B)** analysis.

### 3.3 Insecticidal activity of Sc-ELNs and miRNAs

As shown in **Figure 4A**, Sc-ELNs caused substantial mortality in FAW larvae, with corrected mortality approaching 50% at 24 h. Sc-ELNs also inhibited Sf9 cell proliferation by 48.03% in the MTT assay (**Figure 4B**). Based on small RNA sequencing of Sc-ELNs, five double-stranded miRNA agomirs were synthesized and their effects on FAW larvae were assessed. Among these, the miR-166a-3p agomir and miR-396b-5p produced the strongest effects on mortality and larval growth. On the contrary, other miRNAs did not show significant and sustained insecticidal effects (**Supplementary Figures S1**–**S3**). As shown in **Figures 5A–D**, injection of 100 μM miR-166a-3p agomir increased FAW mortality over time, with significantly higher mortality than the control group. Corrected mortality rate exceeded 50% from day 4 onwards. miR-166a-3p agomir also significantly reduced larval body length. From day 2 to day 5 mean body length in control groups was 2.50, 2.72, 2.75, and 2.66 cm, respectively which were 0.4, 0.39, 0.41, and 0.63 cm greater than the miR-166a-3p agomir-treated group. In contrast, miR-166a-3p agomir did not significantly affect larval weight or food intake overall.

**Figure 4:**
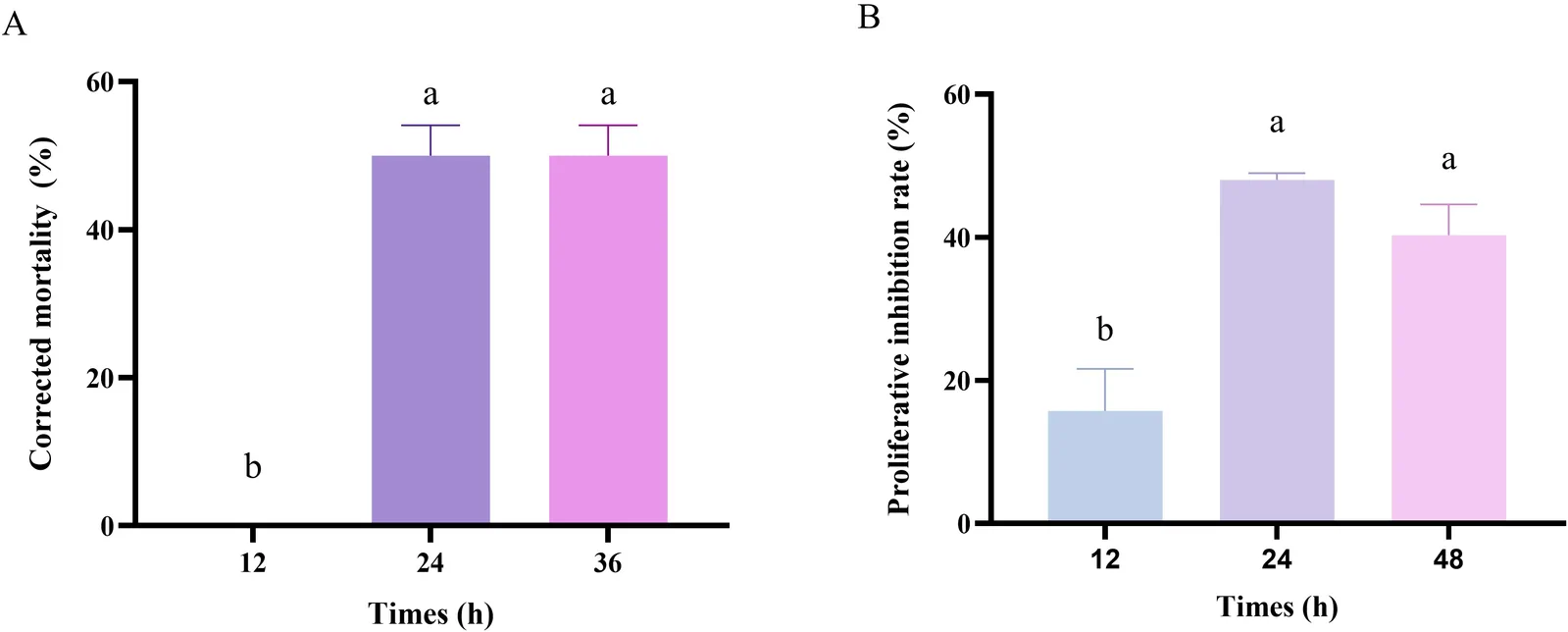
Insecticidal activity of Sc-ELNs *in vivo* **(A)** and *in vitro* **(B)**. Different lowercase letters above columns indicate the statistical differences with p<0.05 via Tukey’s multiple-comparison test analysis.

**Figure 5:**
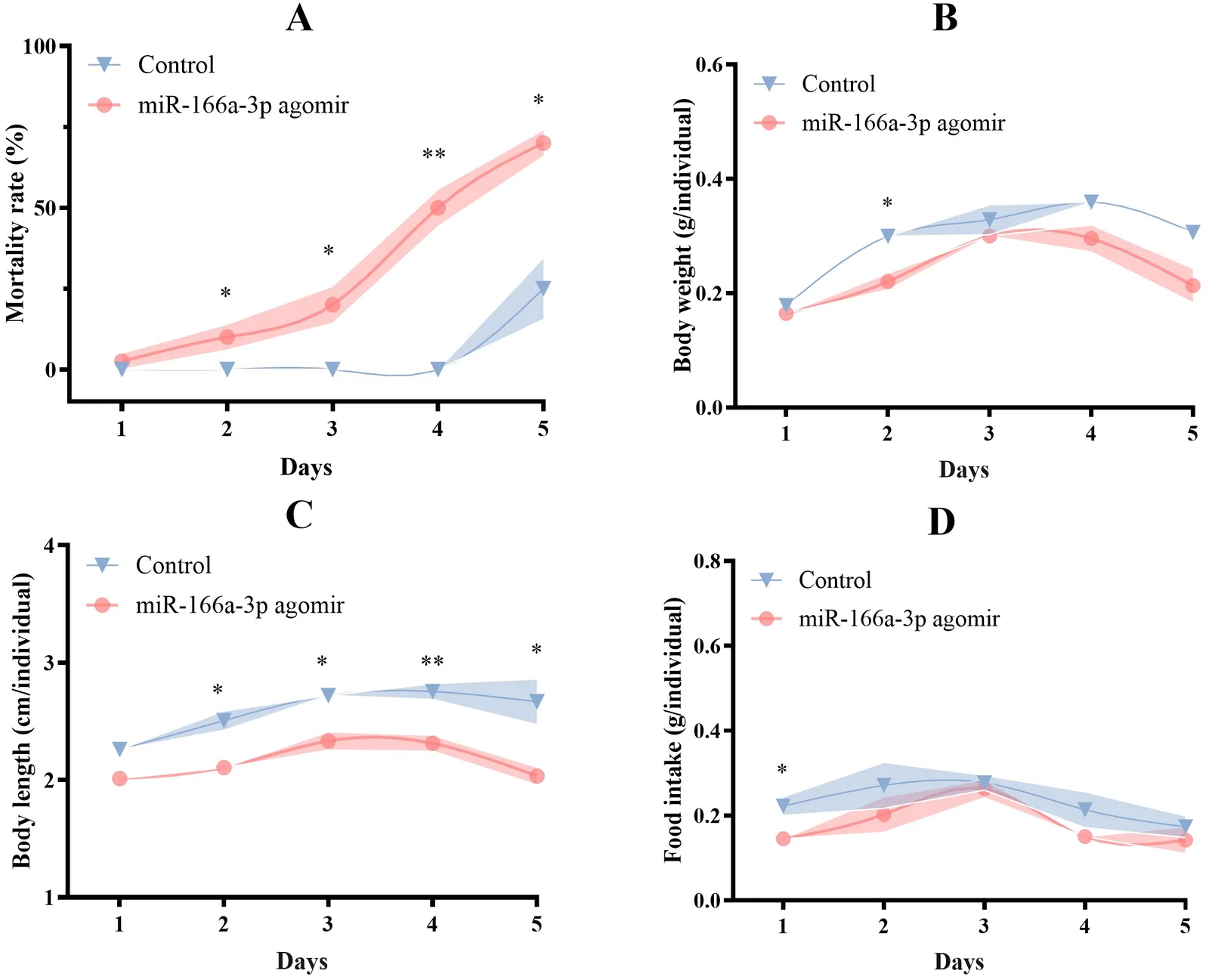
Effects of miR-166a-3p agomir on mortality **(A)**, body weight **(B)**, body length **(C)** and food intake **(D)** on FAW. Experiments were performed in four independent replicates, with 10 larvae per replicate. Asterisks indicate significant differences from the control as determined by Student’s t-test (*p<0.05, **p<0.01).

As shown in **Figures 6A–D**, miR-396b-5p induced a maximum corrected mortality of 53.33% on day 5, which was significantly higher than control. miR-396b-5p also impaired larval growth: on days 4 and 5, mean larval weight and body length in the control group exceeded those of the treatment group by 0.1g and 0.31cm, 0.07g and 0.45cm, respectively. Food intake did not differ significantly between miR-396b-5p and the control groups overall. These results demonstrate that miR-166a-3p agomir and miR-396b-5p have measurable insecticidal and growth-inhibitory effects in FAW, with limited effects on food intake under the conditions tested.

**Figure 6:**
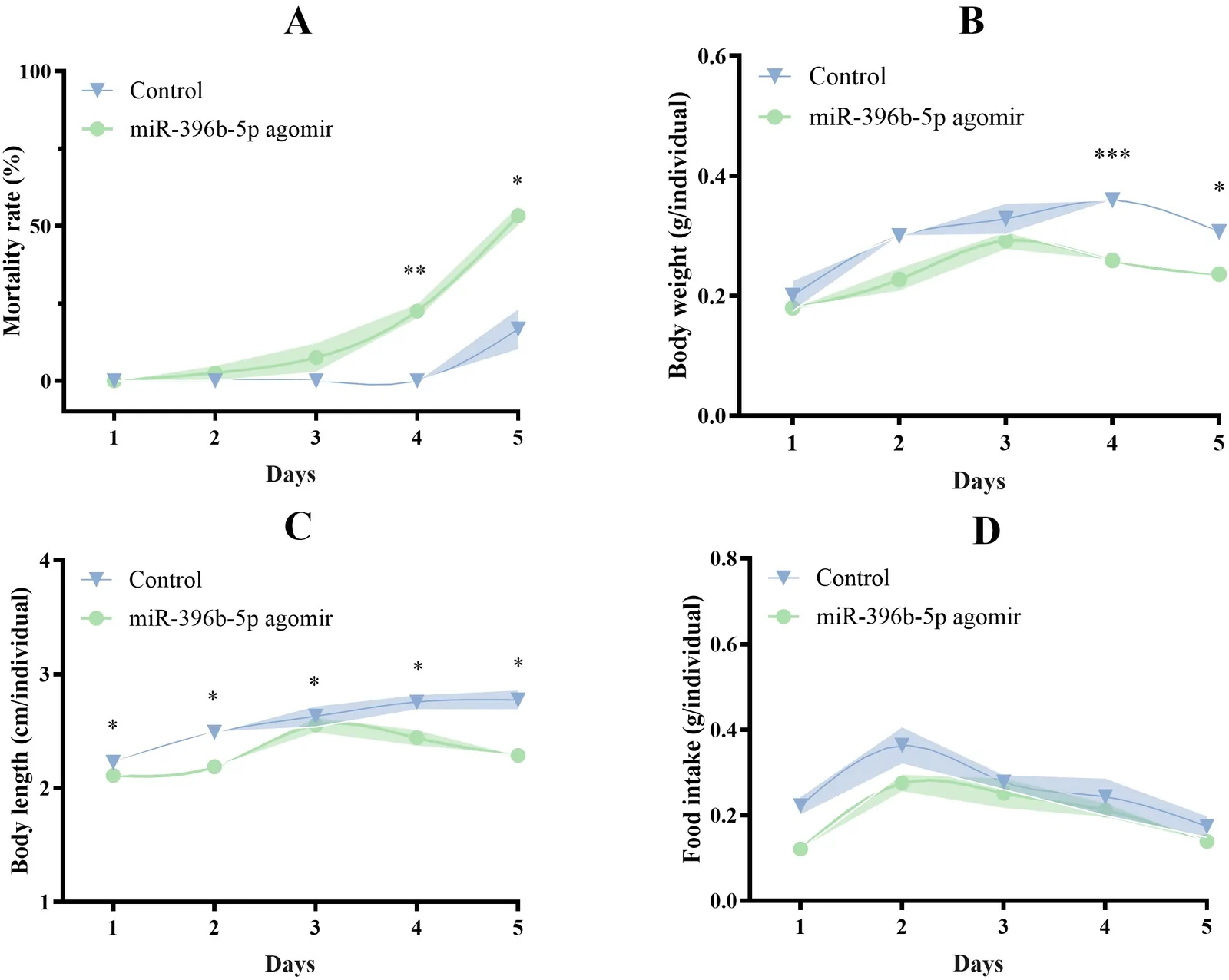
Effects of miR-396b-5p on mortality **(A)**, body weight **(B)**, body length **(C)** and food intake **(D)** of FAW. Experiments were performed in four independent replicates, with 10 larvae per replicate. Asterisks indicate significant differences from the control as determined by Student’s t-test (*p<0.05, **p<0.01, ***p<0.001).

### 3.4 Damaging effect of miR-166a-3p agomir on the midgut of FAW

H&E staining showed that miR-166a-3p agomir treatment was associated with contraction of the circular muscle layer and disruption of the epithelial layer, including loss of columnar cells and regenerative cells along the luminal side of the midgut (**Figure 7**). TEM analysis further indicated reduced cytoplasmic electron density in the major midgut epithelial cell types, consistent with decreased cytoplasmic content (**Figure 8**). Swollen mitochondria and autophagosome-like structures were also observed in columnar and goblet cells following miR-166a-3p agomir treatment (**Figure 8**). TUNEL assay showed a marked increase in nuclear fluorescence in the miR-166a-3p agomir group compared with controls, consistent with increased DNA fragmentation (**Figure 9**). Immunohistochemistry further showed that the expression level of pro-apoptotic protein Bax in columnar cells and regenerative cells increased after treatment with miR-166a-3p agomir (**Figure 10**). These results suggest that the miR-166a-3p agomir midgut epithelial integrity is associated with apoptosis- and autophagy-related cellular changes in midgut cells.

**Figure 7:**
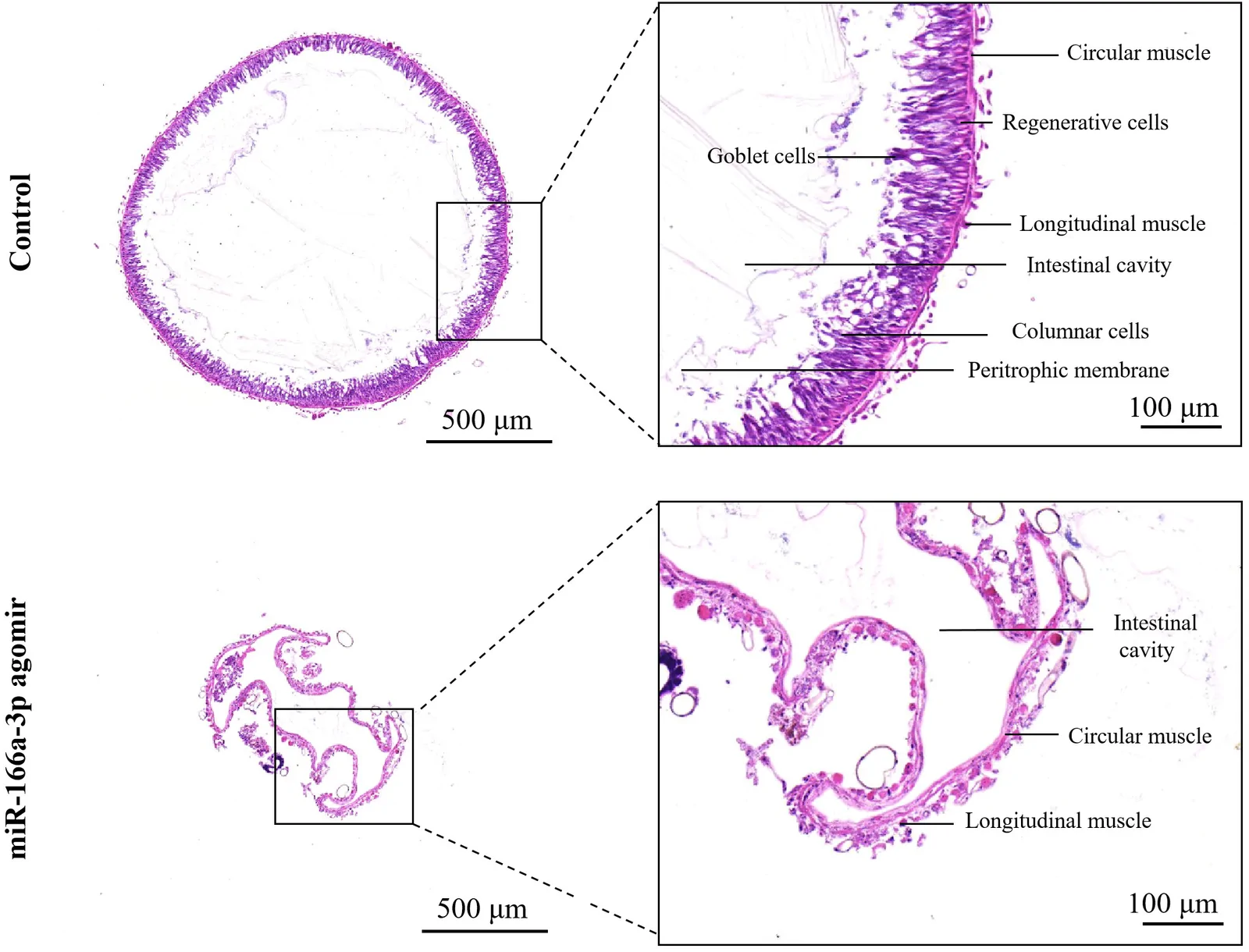
Morphology analysis of the midgut by hematoxylin-eosin stain after miR-166a-3p agomir treatment.

**Figure 8:**
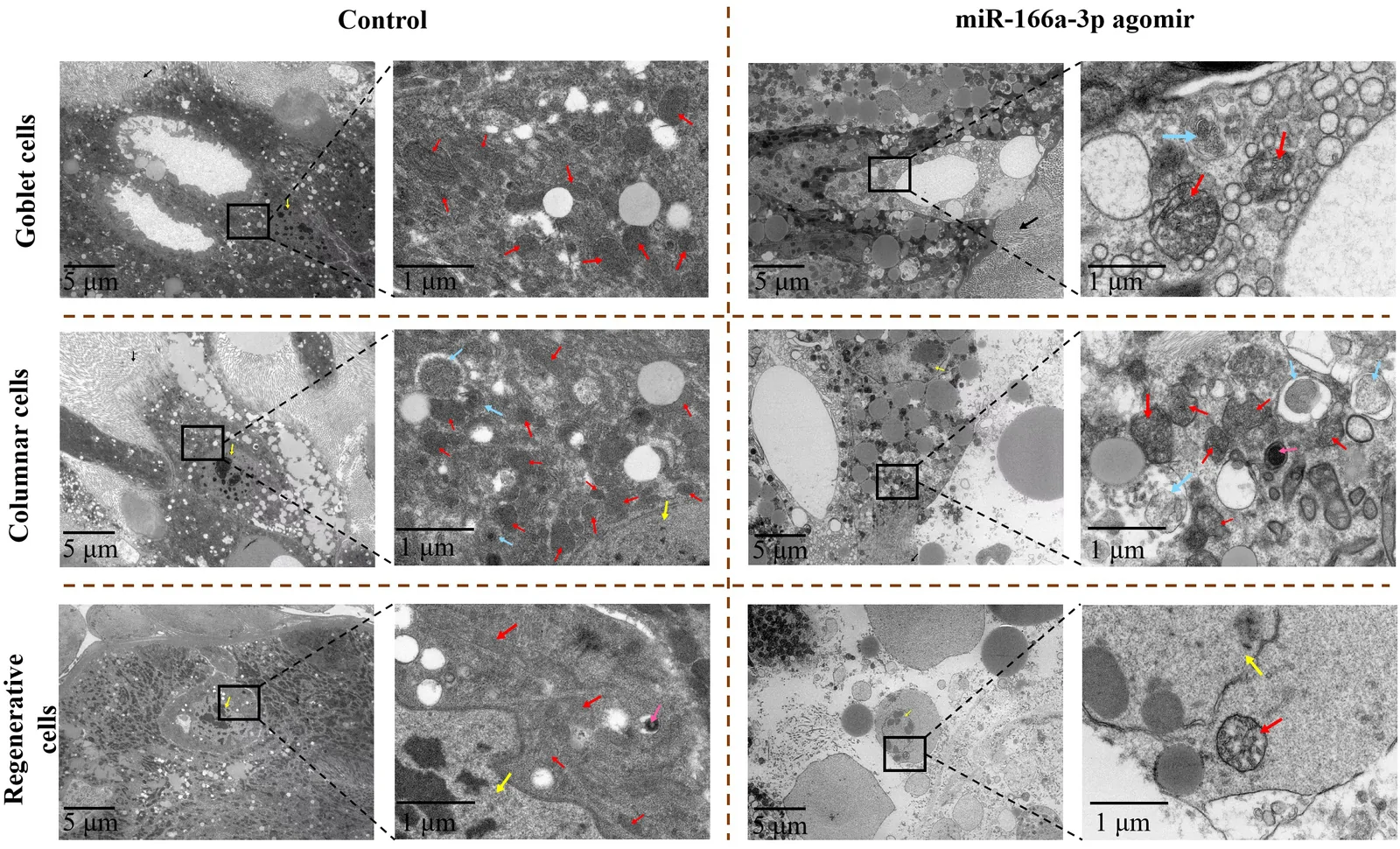
Ultrastructural analysis of the midgut by TEM after miR-166a-3p agomir treatment. Yellow arrows (nuclei), red arrows (mitochondria), black arrows (microvilli), blue arrows (autophagosomes), and pink arrows (autolysosomes).

**Figure 9:**
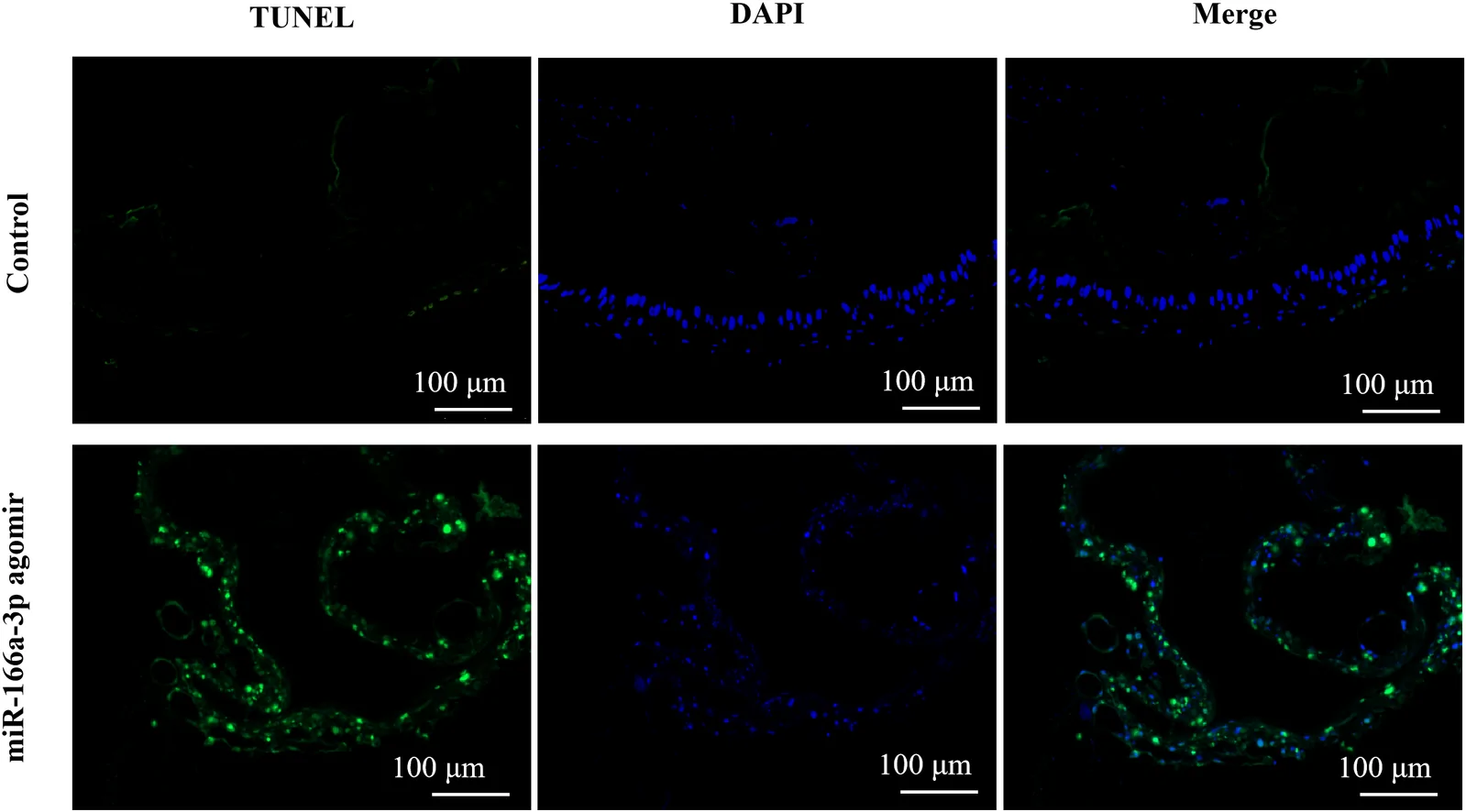
Apoptosis of the midgut by TUNEL analysis after miR-166a-3p agomir treatment.

**Figure 10:**
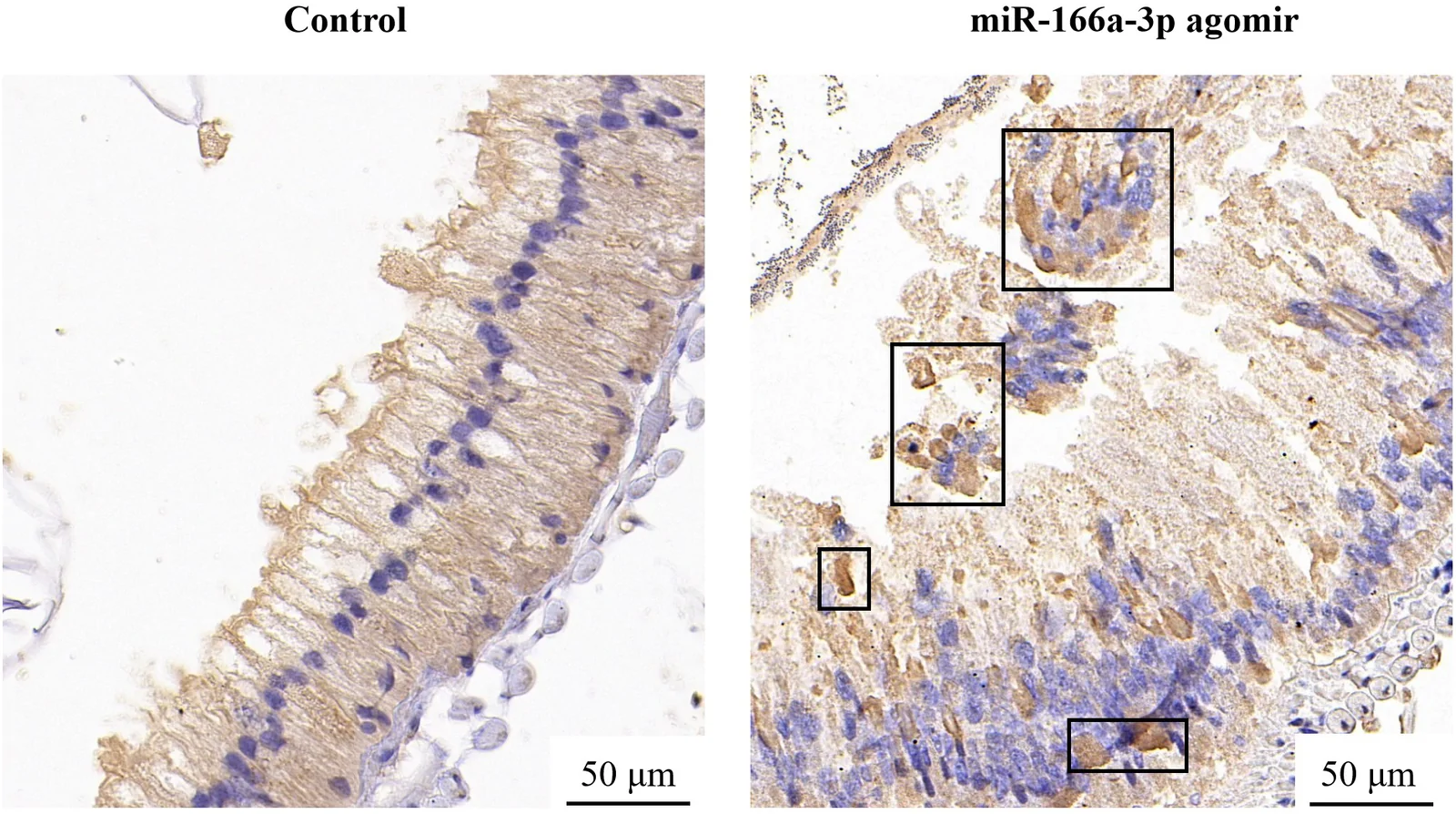
The expression level of pro-apoptotic protein Bax in midgut by immunohistochemistry assay after miR-166a-3p agomir treatment.

### 3.5 Effect of miR-166a-3p agomir on FAW gut bacteria

More than 7 Gb of nonhost clean data with a Q30 score above 94% were obtained for individual samples, confirming data quality and suitability for downstream analysis (**Supplementary Table S2**). A total of 379,514 bacterial genes were predicted across samples and annotated using the Micro_NR database (**Figure 11A**). At multiple taxonomic ranks, miR-166a-3p agomir treatment was associated with reduced relative abundance of Pseudomonadota (phylum), Gammaproteobacteria (class), Enterobacterales (order), Enterobacteriaceae (family), and the genera *Klebsiella* and *Enterobacter* (**Figures 11B–F**). In addition, miR-166a-3p agomir altered the abundance of several less abundant taxa, including increased relative abundance of Syntrophobacterales and Veillonellaceae and decreased abundance of *Asaia, Acidomonas*, and *Gluconacetobacter* (**Figure 11G**).

**Figure 11:**
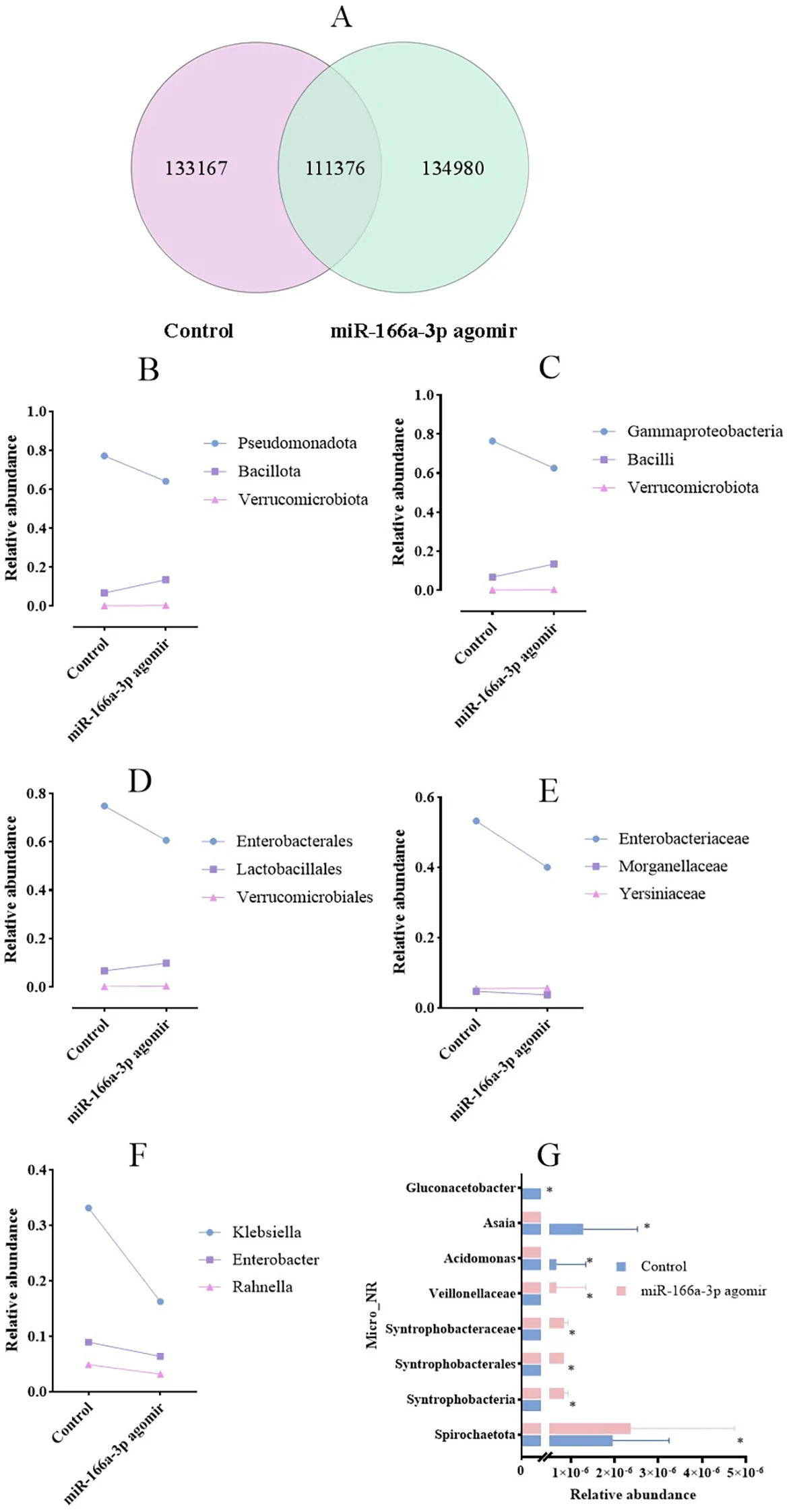
Bacterial identification and abundance in FAW after miR-166a-3p agomir treatment. **(A)** Venn diagram of total identified bacterial genes. The changes in the abundance of the top five bacteria at the phylum **(B)**, class **(C)**, order **(D)**, family **(E)**, genus **(F)** level. **(G)** Bacteria with significant changes at different classification levels. Asterisks indicate significant differences from the control as determined by Student’s t-test (*p<0.05).

Functional annotation against the CAZy database identified 13,239 carbohydrate-active enzyme (CAZyme) genes, predominantly including glycoside hydrolases (GH, 5932 genes) representing the largest group (**Figure 12A**). Compared with controls, miR-166a-3p agomir treatment was associated with reduced relative abundance of GH and glycosyltransferases (GT) at CAZy level 1, AA9 and GH89 at level 2 (**Figures 12B, C**). At the enzyme commission (EC) level, several glycosyltransferase-related functions were enriched in the miR-166a-3p agomir group (**Figure 12D**).

**Figure 12:**
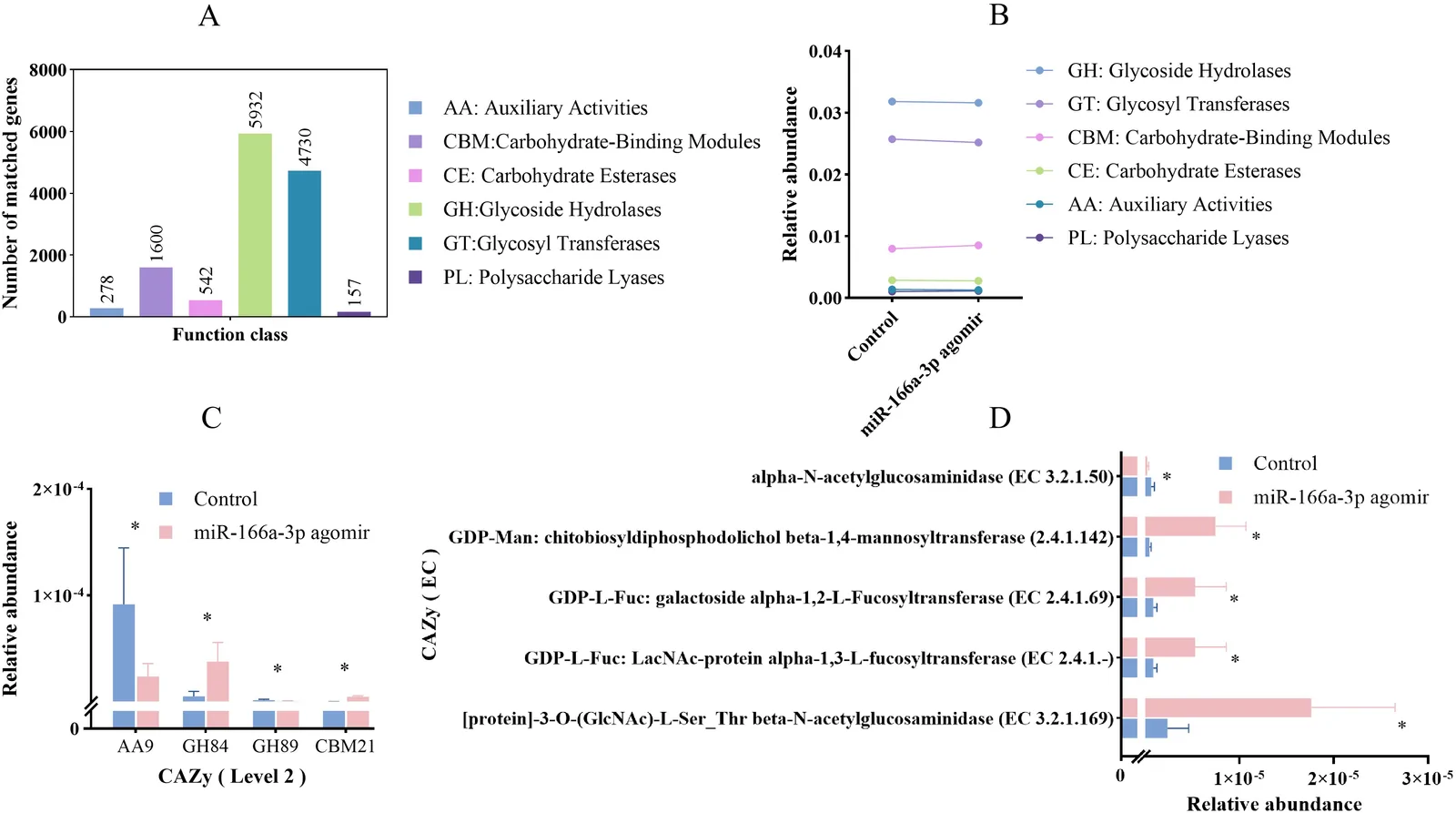
CAZy analysis of bacterial functional genes in FAW after miR-166a-3p agomir treatment. **(A)** Gene annotation and classification. The changes in the abundance of bacterial functional genes at the levels 1 **(B)**, Functional genes with significant changes at level 2 **(C)** and enzyme commission **(D)** level. Asterisks indicate significant differences from the control as determined by Student’s t-test (*p<0.05).

KEGG analysis showed that most predicted genes fell within Environmental Information Processing and Metabolism categories, with carbohydrate metabolism being the largest group (10,185 genes) (**Figure 13A**). Relative abundance of metabolism -related pathways was reduced at level 1, including carbohydrate, amino acid and energy metabolism at level 2, as well as ko02010 (ABC transporters) at level 3 (**Figures 13B–D**). These results suggest that miR-166a-3p agomir treatment is associated with reduced microbial capacity for carbohydrate utilization and nutrient transport. Several level 3 pathways increased in relative abundance, including ko04024 (cAMP signaling), ko04210 (apoptosis), ko04014 (Ras signaling), and ko04071 (sphingolipid signaling) (**Figure 13E**). These pathways are commonly involved in cellular stress responses. At the KO level, a total of 14 metabolism-related enzymes were significantly reduced after miR-166a-3p agomir treatment (**Figure 13F**). Among them, at least four functional modules were directly associated with the tricarboxylic acid (TCA) cycle or its upstream substrate-supplying pathways. Overall, these data indicate a shift in both the taxonomic composition and predicted metabolic functions of the FAW gut microbiota following miR-166a-3p agomir treatment.

**Figure 13:**
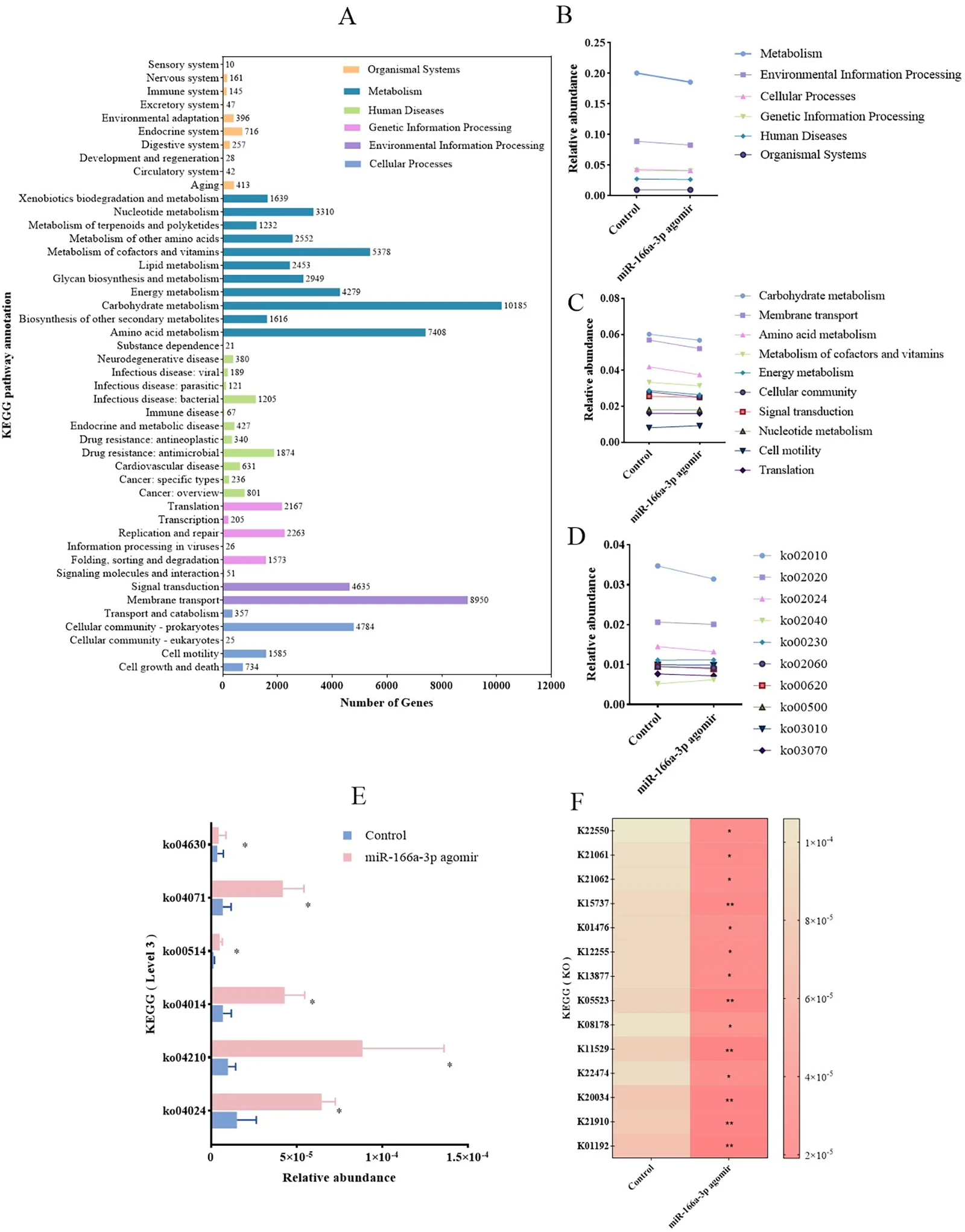
KEGG analysis of bacterial functional genes in FAW after miR-166a-3p agomir treatment. **(A)** KEGG pathway annotation and classification. The changes in the abundance of bacterial functional genes at the levels 1 **(B)**, levels 2 **(C)** and levels 3 **(D)**. Functional genes with significant changes at level 3 **(E)** and KO **(F)** level. Asterisks indicate significant differences from the control as determined by Student’s t-test (*p<0.05, **p<0.01).

## 4 Discussion

*S. chamaejasme* has been reported possess insecticidal activity against FAW and may represent a source of plant-derived control agent. Previous phytochemical studies have suggested that biflavonoid are major constituents of *S. chamaejasme.* However, we have newly identified plant-derived extracellular vesicle-like nanoparticles from *S. chamaejasme* (Sc-ELNs) are also an additional bioactive component. Sc-ELNs increased FAW mortality and effectively inhibited proliferation of the Sf9 cells. Among the miRNAs identified in Sc-ELNs, a synthetic miR-166a-3p agomir significantly increased FAW mortality and was associated with midgut damage, as well as with shifts in gut microbial community composition and predicted functional profiles. These findings expand the repertoire of candidate bioactive agents from *S. chamaejasme* and support further investigation into Sc-ELNs and their miRNA cargo as potential contributors to its insecticidal activity.

Several established methods are available for ELNs isolation, including differential ultracentrifugation, density gradient centrifugation, polyethylene glycol (PEG) precipitation, size-exclusion chromatography, and optimized hybrid protocols (Yin et al., 2024). For example, ultracentrifugation has been used to isolate ELNs from highland barley seedlings (Sha et al., 2024a), and PEG precipitation has also been reported for isolating ELNs from ginger and tartary buckwheat seeds (Zhang et al., 2024). In this study, both ultracentrifugation and PEG precipitation were successfully used, for the first time, to prepare Sc-ELNs from *S. chamaejasme*. Notably, Sc-ELNs prepared via PEG precipitation showed a larger mean particle size than those obtained by ultracentrifugation. A similar pattern was reported by Zhang et al. (2024), who found that extracellular vesicles extracted from tartary buckwheat using PEG had a larger mean particle size (average 182.8 nm) than those obtained using other methods (average 141.8 nm) (Liu et al., 2024). This difference may be due to PEG co-precipitating a broader particle population, together with protein contaminants. Kim et al. (2015) established a PEG/dextran (DEX) aqueous two-phase system for animal exosome extraction, which reduced protein contamination compared with conventional PEG methods, as the exosomal phospholipid bilayer preferentially partitioned into the DEX-rich lower phase, leaving most impurities in the PEG phase. Therefore, relying solely on particle size as a criterion for identifying extracellular vesicles has limitations. Including zeta potential measurements would provide valuable information on nanoparticle surface charge, colloidal stability, and potential for cellular uptake, thereby improving the overall characterization of the ELNs. Although purity can remain a limitation, PEG precipitation offers clear advantages in yield. In our study, using the same starting material, PEG precipitation produced nearly 200-fold more Sc-ELNs than ultracentrifugation. In addition, PEG-based isolation enables higher throughput at lower cost with simpler operation, making it more suitable for scalable production.

ELN have been reported to exert cross-species regulatory effects. In this study, Sc-ELNs from *S. chamaejasme* showed insecticidal activity against FAW *in vitro* and *in vivo*. Plants serve as the primary food source for insects, which providing an effective pathway for ELNs to enter and function in insects. ELNs can contain proteins, lipids, nucleic acids, and plant-specific metabolites. Limited studies have shown that ELN proteins may be involved in cross-species communication and signaling (Sha et al., 2024b). Lipids are also key components extracellular vesicles. In mammalian systems, lipids have been studied extensively for their roles in vesicle biogenesis, release, immune modulation, targeting, and signaling (Sha et al., 2024b). ELN membranes contain phospholipids such as phosphatidylethanolamine and phosphatidylcholine. The relative proportions of these lipids can vary across plant sources, which may affect biodistribution and tissue targeting. For example, Aimaletdinov and Gomzikova (2022) analyzed extracellular vesicle distribution in animals under different dietary conditions, highlighting that administration route and vesicle composition can strongly influence biodistribution.

ELNs also contain diverse non-coding RNAs including miRNAs, lncRNAs, circRNAs, siRNAs, which may contribute to intercellular and cross-species communication. Among these, miRNAs were the highly conserved small RNAs of approximately 20–24 nucleotides that guide the RNA-induced silencing complex (RISC) to cleave complementary mRNAs or repress their translation, thereby regulating gene expression. For example, Liu et al. (2022) reported that the miR6300 mimic from ELNs derived from tartary buckwheat promoted the growth of the human-associated bacterial strains *Escherichia coli* and *Lactobacillus rhamnosus*. In a recent study, honey-derived ELNs containing miRNA4057 were reported to downregulate IL-6 and TNF-α expression in mammals via specific signaling pathways (Chen et al., 2021). In our study, miRNAs identified in Sc-ELNs were predicted to target genes of *Drosophila* related to energy metabolism and programmed cell death, which revealed its potential for cross-species regulation. As a model organism of insects, fruit flies have a clear genome background and complete annotations, and the predicted results have high reliability and universality. Furthermore, we validated the insecticidal activity of miRNA agomirs using FAW, and the results showed that the synthetic miR-166a-3p agomir and miR-396b-5p agomir reduced FAW survival and impaired larval growth. However, most FAW genes lack well-annotated 3′ UTRs, making it difficult to accurately predict the binding sites of miR-166a-3p and miR-396b-5p on mRNAs of FAW, nor is it possible to compare whether the binding sites of the two miRNAs in FAW are conserved with *Drosophila*. To address this limitation, we screened target genes previously identified in fruit flies and found that *AnxB10* and *Snx27*, both targets of miR-166a-3p, are conserved in FAW (**Supplementary Figures S4**, **S5**). *AnxB10* belongs to the annexin family and is involved in cell membrane repair and fusion, as well as apoptosis (Mitsutoshi and Susan, 2024; Reutelingsperger and Van Heerde, 1997). *Snx27* binds to phosphatidylinositol-3-phosphate, a key signaling molecule in the PI3K pathway, and participates in cellular autophagy and proliferation (Vergne and Deretic, 2010).

Together, these findings suggest that miRNA cargo may contribute to Sc-ELNs bioactivity and raise the possibility that synthetic miRNA agomirs could be explored as a more scalable alternative to vesicle-based formulations. However, cross-species effects of plant-derived miRNAs remain debated, particularly with respect to stability, delivery, and whether physiologically relevant concentrations can be achieved *in vivo*. Exosomal encapsulation has been proposed to protect plant miRNAs from degradation in the gastrointestinal tract (Qin et al., 2022), but the stability and bioavailability of different ELNs across insect gut environments remain uncertain and may limit practical application. Therefore, it is necessary to combine fluorescent labeling, RT-qPCR, and luciferase reporter gene detection techniques in future research to assist in determining the actual uptake of miRNA and direct mutual evidence between miRNA and predicted gene targets. As far as this study is concerned, the current Sc-ELNs associate miRNA target genes are mainly predicted based on bioinformatics. The actual interaction and regulatory effects between Sc-ELNs associate miRNA and its target genes in insect still need further confirmation at the molecular level.

Apoptosis and autophagy represent intrinsic programmed cell death responses to cellular stress (Wu et al., 2015; Suganuma et al., 2011). In honey bees, pesticide exposure has been reported to trigger apoptosis in larval midguts, salivary glands, and ovaries as a protective response (Teng et al., 2018). Polyethylene microplastics have also been shown to induce autophagosome formation in midgut of FAW (Fan et al., 2025). In the present study, injection of Sc-ELN miRNAs impaired FAW larval development without significantly reducing food intake, suggesting a potential effect on nutrient utilization. Consistent with other stressors that damage midgut structure and function, miR-166a-3p agomir treatment caused pronounced epithelial disruption in FAW larvae. TUNEL staining revealed increased DNA fragmentation, and TEM showed autophagosome-like structures in midgut epithelial cells. Furthermore, increased Bax immunostaining supported the activation of apoptosis-related responses in the midgut epithelium.

In our study, miR-166a-3p agomir injection not only disrupted the midgut epithelial barrier in FAW, but also altered the composition of gut microbiota, which may represent an additional mechanism contributing to growth inhibition. The gut microbiota is essential for systemic physiology by regulating immunity, nutrient uptake, and metabolism, and directly supports energy homeostasis, maintains gut barrier integrity, and activates mucosal immune defenses (Spiljar et al., 2017). ELN’s targeted delivery system has been proposed allows precise modulation of gut microbiota composition, effectively altering key genera such as *Bacteroides* and *Lactobacillus*. Consistent with this, Teng et al. (2018) demonstrated that ginger-derived lipid nanoparticles (GELN) are internalized by gut microbes, promoting *Lactobacillus* and *Bacteroides* growth while suppressing *Clostridium*. In our study, miR-166a-3p agomir treatment reduced the relative abundance of several acetic acid bacteria in the FAW gut, including *Asaia*, *Acidomonas*, and *Gluconacetobacter*. *Asaia* is an important component of the gut microbiota in *Anopheles stephensi*, and loss of *Asaia* symbionts has been reported to delay larval development (Chouaia et al., 2012). Meanwhile, miR-166a-3p agomir treatment was associated with reduced relative abundance of the genera *Klebsiella* and *Enterobacter*, which are commonly reported as gut-associated taxa that contribute to carbohydrate utilization and may influence host growth and defense (Li et al., 2025; Zhang et al., 2021).

Furthermore, functional annotation of gut bacterial genes showed that miR-166a-3p agomir treatment reduced the relative abundance of various glycoside hydrolases. These changes are consistent with the potential decrease in the ability of microorganisms to degrade complex polysaccharides, including cellulose (**Figure 14**). Ultimately, this pattern may affect the utilization of dietary carbon sources by host midgut epithelial cells via microorganisms. In addition, several KOs linked to microbial energy metabolism decreased in abundance. Reduced K21910 suggests a reduced capacity for glycolytic intermediate formation, which may limit pyruvate production and downstream acetyl-CoA supply to the TCA cycle. Decreases in proline and hydroxyproline metabolism associated enzymes (K22550, K21061, K21062) may reduce replenishment of α-ketoglutarate while reduced K15737 may further limit conversion of glutarate to α-ketoglutarate, reducing anaplerotic inputs into the TCA cycle. Finally, decreased K22474 may reflect impaired electron transport and NAD^+^ regeneration, consistent with reduced efficiency of terminal energy conversion.

**Figure 14:**
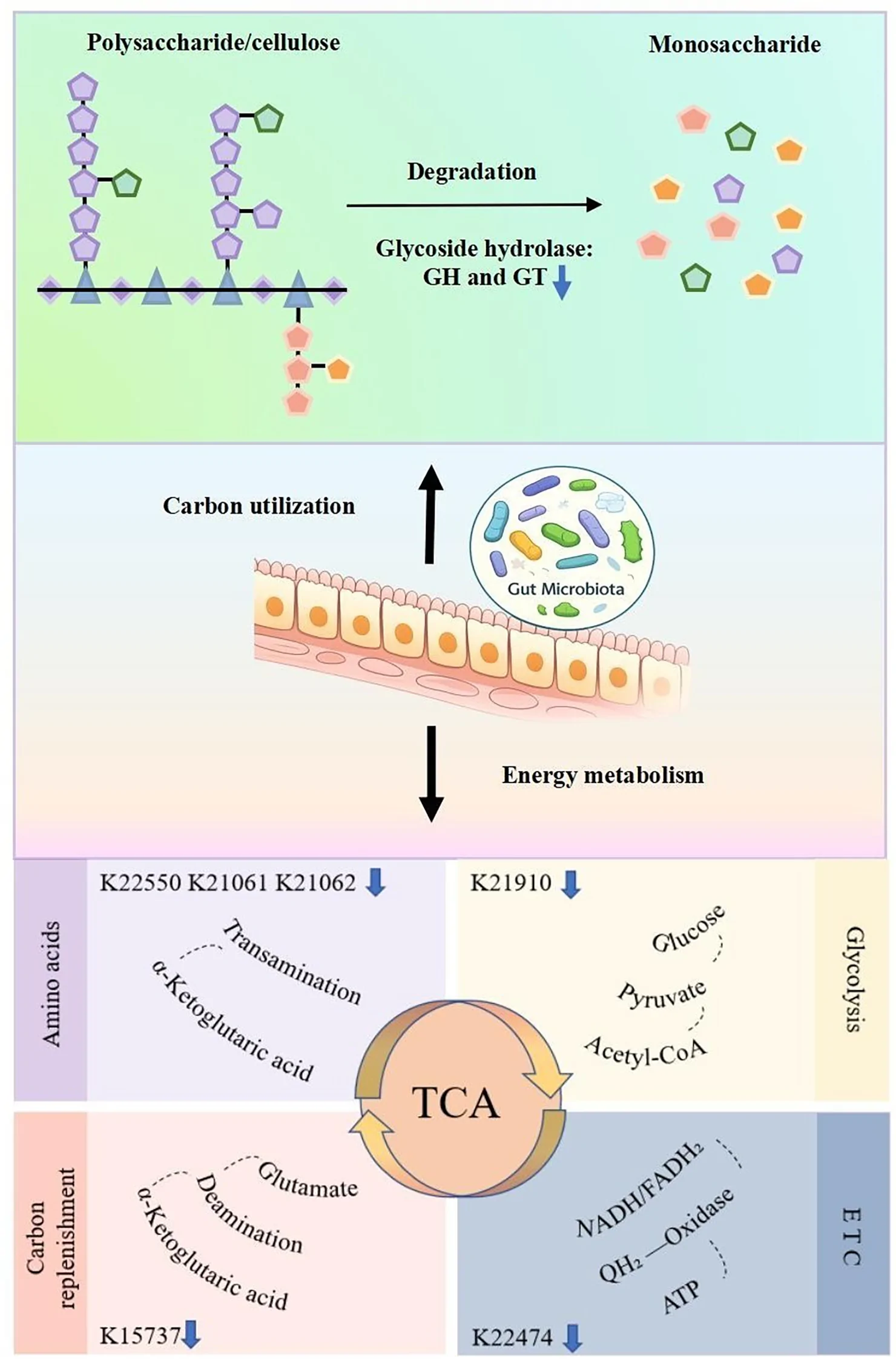
Schematic diagram of miR-166a-3p agomir’s mechanism for inhibiting carbon source utilization and energy metabolism in gut bacteria of FAW.

Our findings indicate that Sc-ELNs and their miRNAs cargo are associated with insecticidal effect in FAW. In particular, miR-166a-3p agomir treatment was involved in midgut epithelial cells apoptosis. miR-166a-3p agomir also disrupted gut microbiota, including reduced abundance of several taxa linked to nutrient utilization and energy acquisition. Collectively, these results support a potential cross-species regulatory role of miRNAs in Sc-ELNs for FAW, and provide a novel strategy for discovering potential insecticidal components in plants.

## Data Availability

The Illumina sequencing data has been deposited in the NCBI Sequence Read Archive (SRA).The accession number is PRJNA1532772.
