# Supplementary material for: The miR-166a-3p in exosome-like nanoparticles from Stellera chamaejasme induce insecticidal effects in fall armyworm by disrupting the gut barrier and microbiota
Source: Front Plant Sci. 2026 Sep 30;17:1902418. doi: 10.3389/fpls.2026.1902418 (PMC13661944; doi:10.3389/fpls.2026.1902418)
Supplement: Supplementary file 1 [file SupplementaryFile1.docx]

**Supplementary Documents**

**Table S1.** Summary of sRNA sequencing data.

| Sample | Total reads | Q30 (%) | Clean reads | GC content (%) | Total sRNA | Mapped sRNA | “+” Mapped sRNA | “-” Mapped sRNA | Mapped mature miRNA |
| --- | --- | --- | --- | --- | --- | --- | --- | --- | --- |
| Sc-ELNs | 11,487,458 | 97.05 | 10,796,003 | 55.49 | 3,203,956 | 155,196 | 108,678 | 46,518 | 5 |

**Table S2.** Summary of gut bacteria metagenomics data.

| Sample | Raw Base (G) | Clean Base (G) | Clean Q30 (%) | Clean GC (%) | Effective (%) | Non-host Base (G) | Average length after assembly (bp) |
| --- | --- | --- | --- | --- | --- | --- | --- |
| Control 1 | 7.73 | 7.69 | 94.63 | 51.37 | 99.54 | 7.24 | 1027.95 |
| Control 2 | 9.91 | 9.85 | 94.88 | 50.48 | 99.4 | 7.25 | 1102.39 |
| Control 3 | 26.11 | 25.98 | 96.58 | 39.5 | 99.48 | 7.74 | 913.50 |
| miR-166a-3p agomir  Treatment 1 | 20.68 | 20.55 | 96.58 | 40.36 | 99.36 | 7.2 | 956.88 |
| miR-166a-3p agomir  Treatment 2 | 20.34 | 20.22 | 96.48 | 40.65 | 99.44 | 8.1 | 1176.63 |
| miR-166a-3p agomir  Treatment 3 | 33.02 | 32.8 | 96.11 | 39.3 | 99.33 | 8.36 | 1063.23 |

**Figure S1.** Effects of miR-159a-3p agomir on mortality (A), body weight (B), body length (C) and food intake (D) on FAW. Experiments were performed in three independent replicates, with 15 larvae per replicate. The data are presented as the mean ± standard error. Asterisks indicate significant differences from the control as determined by Student’s t-test (*p＜0.05, **p＜0.01).

**Figure S2.** Effects of miR-159b-3p agomir on mortality (A), body weight (B), body length (C) and food intake (D) on FAW. Experiments were performed in four independent replicates, with 10 larvae per replicate. The data are presented as the mean ± standard error. Asterisks indicate significant differences from the control as determined by Student’s t-test (*p＜0.05, **p＜0.01).

**Figure S3.** Effects of miR-159c-3p agomir on mortality (A), body weight (B), body length (C) and food intake (D) on FAW. Experiments were performed in four independent replicates, with 10 larvae per replicate. The data are presented as the mean ± standard error. Asterisks indicate significant differences from the control as determined by Student’s t-test (*p＜0.05, **p＜0.01).

**Figure S4**. Multiple amino acid sequence alignment of *AnxB10* orthologs between *D. melanogaster* and *S. frugiperda*. Black font indicates highly conserved residues (relative entropy ≥ 2 bits). Red font denotes insertion events and mutation.

**Figure S5**. Multiple amino acid sequence alignment of *Snx27* orthologs between *D. melanogaster* and *S. frugiperda*. Black font indicates highly conserved residues (relative entropy ≥ 2 bits). Red font denotes insertion events and mutation.
